# The GeoCitizen-approach: community-based spatial planning – an Ecuadorian case study

**DOI:** 10.1080/15230406.2014.890546

**Published:** 2014-03-10

**Authors:** Karl Atzmanstorfer, Richard Resl, Anton Eitzinger, Xiomara Izurieta

**Affiliations:** ^a^ Department of Geoinformatics – Z_GIS, Doctoral College GIScience, University Salzburg, Hellbrunnerstrasse 34, 5020Salzburg, Austria; ^b^ Universidad San Francisco de Quito, Av. Diego de Robles y Via Interoceánica, Cumbaya, Ecuador; ^c^ Decision and Policy Analysis Program (DAPA), International Center for Tropical Agriculture (CIAT), CIAT, A.A. 6713, Cali, Colombia

**Keywords:** geospatial web platforms, social media, Public Participatory GIS (PPGIS), empowerment, community-based spatial planning

## Abstract

Over the last years, geospatial web platforms, social media, and volunteered geographic information (VGI) have opened a window of opportunity for traditional Public Participatory GIS (PPGIS) to usher in a new era. Taking advantage of these technological achievements, this paper presents a new approach for a citizen-orientated framework of spatial planning that aims at integrating participatory community work into existing decision-making structures. One major cornerstone of the presented approach is the application of a social geoweb platform (the *GeoCitizen* platform) that combines geo-web technologies and social media in one single tool allowing citizens to collaboratively report observations, discuss ideas, solve, and monitor problems in their living environment at a local level. This paper gives an account of an ongoing participatory land-zoning process in the Capital District of Quito, Ecuador, where the *GeoCitizen* platform is applied in a long-term study.

## Volunteered Geographic Information, Web 2.0, participation and empowerment

In the last decade, technological achievements like GPS, smartphones, virtual globes, and a variety of other Web 2.0 tools have facilitated the dissemination of spatial information and the collection of crowd-sourced (spatial) data (Sui, Elwood, and Goodchild 2013). Beyond the spatial domain, social media such as Facebook or Twitter are increasingly available for a wide range of users, even in emerging societies of developing countries (Evans-Cowley 2010). These rapidly evolving technologies have brought new perspectives for redefining participatory spatial planning and public administration. The combination of both – crowd-sourced spatial data and discussion frameworks based on the concepts of social media – creates so far unknown possibilities for communities and citizens to participate in planning processes aiming at empowering them to better manage their living environments.

An increasing number of consumers of spatial data are not only using, but also producing spatial data (Fischer 2012). Such “prosumers” of spatial data can increasingly resort to freely available cartographic data like base-maps provided by non-profit (collaborative mapping) initiatives such as OpenStreetMap (Goodchild 2010). This creation, sharing, and usage of spatial data by laypersons is termed volunteered geographic information (VGI) and is a current issue of in-debt discussions in scientific literature (Goodchild 2007; Elwood 2008; Goodchild 2008; Blaschke and Strobl 2010; Rosser and Morley 2010). User-generated spatial data is a major component of a recent area of research in GIScience that arose as a result of the advent of the Web 2.0: “Neogeography”. Turner (2006) defines “Neogeography” as a set of techniques and tools that fall outside the realm of traditional GIS, such as mapping Association of Independent Professionals of Tumbaco (*Agrupación de Profesionales Independientes de Tumbaco* [APIs]) (e.g. Google Maps), geotagging, or new data formats (e.g. KML, GPX) used for personal and rather colloquial activities by a nonexpert group of users (Turner 2007). Its popularity can be credited to the ability to communicate and share data through simple, freely available tools that can be learnt quickly and effectively without immersion in professional activities (Hudson-Smith et al. 2009).

The term “Geo(spatial) web” refers to the use of the internet to deliver geographic information and maps (Haklay, Singleton, and Parker 2008) or – in other words – to the global collection of applications, services, and data that supports the use of geographic information on the web (López, Béjar, and Zarazaga Soria 2012). As key applications of the “Geo(spatial) web”, geospatial web platforms allow the processing of crowd-sourced spatial data and facilitate laypersons to collect, analyze, and share spatial data helping them to identify problems in their habitat in a transparent and traceable manner, for example (Haklay 2010; Bednarz and Kemp 2011). Besides mapping tools, geospatial web platforms may integrate (video) blogs, RSS-feeds, social network tools (e.g. tweets), discussion forums, widgets, and other applications that allow users to create their own mash-ups, combing online data from multiple sources (Ashley et al. 2009).

In the planning domain, the concept of Public Participatory GIS (PPGIS) emerged in the mid-1990s, referring to the incorporation of nonexpert stakeholders in spatial planning processes (Ghose 2007; Ramasubramanian 2010). McCall and Dunn (2012) define PPGIS as “collaborative and participatory approaches to planning, using GIS” (81). PPGIS link community participation and geographical information in a diversity of social and environmental contexts, involving citizens in decision-making processes (Steinmann, Krek, and Blaschke 2004). For a long time, collaborative mapping initiatives dominated real-world PPGIS applications (Sieber 2006). However, the last decade brought the development of a considerable number of geospatial web platforms that aim at enabling citizen participation in the management of their living environment. These platforms mainly call local governments’ attention to problems regarding the provision of public services, issues related to security, and (public) transportation issues or the environment (e.g. *FixMyStreet* (http://www.fixmystreet.com/), *SeeClickFix* (http://en.seeclickfix.com/) or *ParcScan* (http://www.parkscan.org). For a long time, these applications have been limited to industrialized nations in North America and Europe. However, recently also in the emerging countries of Latin America, new collaborative platforms for citizen engagement have been launched: *CiudadanosActivos* in Cali, Colombia (http://www.ciudadanosactivos.com) or *Deliktum* in Quito, Ecuador (http://www.deliktum.com), to name some examples.

As mentioned above, recently available Web 2.0 techonolgies and their diffusion within society opened up new vistas for participatory planning initiatives. Especially mobile devices have become an important tool for the collection and communication of such data. Aker and Mbiti (2010) examine the growth of mobile phone technology over the past decade and consider its potential impacts upon quality of life in low-income countries. In this respect, Hennig and Vogler (2011) coined the term “social geo-communication” referring to the participation of the public in planning processes supported by Web 2.0 platforms. These platforms provide the ground for “spatial citizens” that are able to “interpret and critically reflect spatial representations, communicate […] and express location-specific opinions with the aid of maps” (Gryl and Jekel 2012, 4). The essential skill of “spatial citizens” is what scholars such as Blaschke and Strobl (2010), Bednarz and Kemp (2011), and Goodchild (2010) refer to as “spatial literacy”. This is the ability of an individual to capture and communicate knowledge in the form of a map, understand and recognize the world as viewed from above, recognize and interpret patterns, know that geography is more than just a list of places on the Earth’s surface, see the value of geography as a basis for organizing and discovering information, and comprehend such basic concepts as scale and spatial resolution (Goodchild 2007).

The idea of “spatial literacy” and “spatial citizens” are an important approach in order to integrate geospatial web platforms into the concept of empowerment. Carver et al. (2001) define empowerment “as the process by which stakeholders identify and shape their lives and the society in which they live through access to knowledge; political processes; and financial, social, and natural resources” (62). By doing so, empowerment of citizens or communities that so far have been excluded from participating in the design and management of their habitat, is an intrinsic aim of any participation initiative. Corbett and Keller (2005) state, that the overarching goal of every PPGIS activity is empowerment, as PPGIS “can be empowering to disadvantaged groups by enabling them to use the language and tools of decision makers and so influence events that affect their lives and local geography” (91). According to Gryl and Jekel (2012), the use of geospatial-web tools is a major factor for democratic negotiation and public participation in the spatial domain referring to the concept of “actualizing citizens” (as opposed to “dutiful citizens”) that act through loose networks using social media and the geospatial-web for communication and interaction. They use digital narratives, which change their relationships to civic knowledge and its components of authority, credibility, production–consumption, and sharing of information.

New technological trends such as geospatial web platforms as well as the use of VGI and their embedding into delicate societal issues of empowerment and public participation trigger research questions in regard to the credibility, quality, and privacy of crowd-sourced data (Haklay 2010). There is a legitimate concern amongst professional GIS-practitioners regarding certainty, accuracy, and quality of spatial data collected by laypersons that might not always meet the quality criteria of data and maps produced by professional cartographers in, for example, public mapping agencies (Goodchild 2008; Crampton 2010; Fischer 2012). Flanagin and Metzger (2008) resort to the term “believability” (according to Hovland, Janis, and Kelley (1953)) when talking about credibility of crowd-sourced spatial information that is composed of two primary dimensions: trustworthiness and expertise. This raises two major questions that have to be considered dealing with spatial data provided by laypersons: Do these data precisely describe reality or are they biased by the person who has created them (*trustworthiness*)? And does this layperson have enough *expertise* in the data acquisition procedure in order to ensure data accuracy?

Furthermore, the publication of VGI on geospatial web platforms might not always meet specific conceptions of privacy that vary throughout different cultures (Torrens 2010). Recently, web platforms such as *RottenNeighbor* (where users have been encouraged to expose “bad” neighbors like sex offenders, see: http://en.wikipedia.org/wiki/Rottenneighbor.com), or *MyBikeLane* (where citizens can report traffic violations like illegally parked cars on bike lanes; see: http://www.mybikelane.com/) have been controversially discussed in public and even raised serious legal concerns.

Moreover, technological and structural limitations for accessing Information and Communications Technology (ICT)-tools must be considered when evaluating the potentials and limitations of these new technological achievements. The selective access to ICT perpetuates exclusive social structures and hence even more exclude marginalized communities from participation (Ghose 2007; Elwood 2008; Haklay 2012). However, the increasing availability of telecommunications infrastructures (especially mobile devices) even in emerging societies and developing countries have helped to close this technological gap (Martin and Corbett 2011). In order to facilitate the use of geospatial web platforms for users with no or just little spatial literacy, application developers and researchers put a strong emphasis on usability issues. User-friendly designed tools and frameworks should also address users that do not have the necessary skills for handling and processing spatial information properly. This is what scholars such as Sui, Elwood, and Goodchild (2013) and Haklay (2010) refer to as “secondary digital divide” that has to be bridged. In order to ensure the “democratization” of spatial data when propagating the use of VGI, it has to be considered that crowd-sourced datasets tend to reflect the characteristics of specific online-communities of interest and do not represent the qualities of a random sample population (Fischer 2012). In this respect, also the issue of ownership of (local) knowledge that is mapped through crowd-sourced data has to be considered, especially within PPGIS projects and initiatives (Haklay, Singleton, and Parker 2008; McCall and Dunn 2012).

Another crucial issue is whether and – if so – to which extent planners resort to participatory initiatives that use social media and other ICT-tools and if these initiatives are turned into real action in the end (Evans-Cowley 2010). Therefore, the conceptual framework in which these technologies are embedded in existing power structures is – besides the issue of usability – an important point to consider when developing geospatial web platforms for public participation and citizen collaboration. The implementation of the concept of “social geo-communication” in participatory spatial planning would demand a framework that fully integrates web-mapping tools and social media in one single platform that is accessible also with mobile devices. However, existing geospatial web-platforms do not yet provide proper tools for discussion and problem solving based on community interaction with the limiting effect that communication between citizens and authorities tends to stay unidirectional. Hence, an increasing number of scholars ask for the amplification of these platforms by adding functionalities that allow citizens to engage in building communities, programing activities, and in finding sound solutions rather than reporting their complaints to central (planning) offices or other institutions (Evans-Cowley 2010; Fu and Sun 2010; Ramasubramanian 2010). This is aimed at with the *GeoCitizen*-framework that is presented in this paper: to describe a new approach for a citizen-orientated framework of spatial planning that aims at integrating participatory community work to existing decision-making processes and applying a social-geoweb platform as essential tool of immediate citizen participation.

## The *GeoCitizen*-approach and platform

The *Geo-Citizen* framework allows citizens and communities to collaboratively report observations, discuss ideas, solve and monitor problems related to spatial planning, as well as the provision of public services and other issues at a local level using a geospatial web-platform. This platform – the *Geo-Citizen* platform – merges geo-web technologies and social media in one single, comprehensive, and interactive tool for participatory spatial planning, community interaction, and citizen collaboration. As citizens are good observers of problems occurring in their communities, this platform enables them to interconnect their observations and perceptions of their living environment on a neighborhood scale with other citizens in the same neighborhood or community. But it also provides an efficient tool to connect themselves to other citizens, communities, and local initiatives outside their immediate neighborhoods or communities that face the same type of problems and that have similar observations or ideas and maybe solution strategies which can be exchanged. This is done in order to find proper solutions for planning-related issues (e.g. infrastructure projects, zoning of land, property, and cadaster-related issues), the provision of public services tailored to the citizens’ needs (public transport, public security, energy and (waste-) water management, public health and educational infrastructure, etc.) or problems that are related to deteriorating environmental conditions (e.g. due to the effects of climate change).

The *GeoCitzen*-platform provides a communication framework that constitutes a social network for citizens and their initiatives that is based on geo-referenced observations of what is affecting their quality of life. By discussing these issues in the public space of the World Wide Web, immanent power relations between citizens, public administration, and other stakeholders of (spatial) decision-making processes should become more transparent, and the flow of information should be boosted. Public authorities should be urged to share information directly to and between the affected citizens and communities. As a consequence, actions of administrative bodies should become more transparent and legitimated by the affected citizens. The proposed *GeoCitizen-*framework establishes an open access entry point for every citizen to identify geographically a problem and connect it to a thematically specific problem-solving process. It enables users to find collaborators in their neighborhood, and to get informed about the spatial context of the problem and the related conflicts. Experts for specific problem domains can be consulted and best practice examples can be exchanged in order to get inspiration from how other communities have solved the same or a similar type of a problem. GIS tools support the construction of problem-focused local knowledge and evidence the collective spatial decision-making process. Issues that so far have been treated in separate frameworks focused on one, single type of a problem (e.g. deteriorating road infrastructure, badly managed recycling of waste) can now be interconnected to an integrative framework that do not treat a problem as a singular issue but as part of a broader (planning) concept. Hence, citizens are empowered to be part of the solution for a problem as they are encouraged to find their proper problem-solving strategies within their communities instead of uniquely resorting to external experts or the action of administrative bodies. This is especially important in regions where government and public administration tends to be inefficient, bureaucratic, and sometimes even corrupt.

The *GeoCitizen*-platform is designed as a cross-platform for web-browsers on desktop computers and mobile devices like smartphones and tablets. It uses the jQuery mobile web-framework (a JavaScript library), the Google Map API, and a PostGres/GIS database as web-application framework. The ArcGIS for Server JavaScript API is used for integrating spatial background layers to the platform.

**Figure 1.**
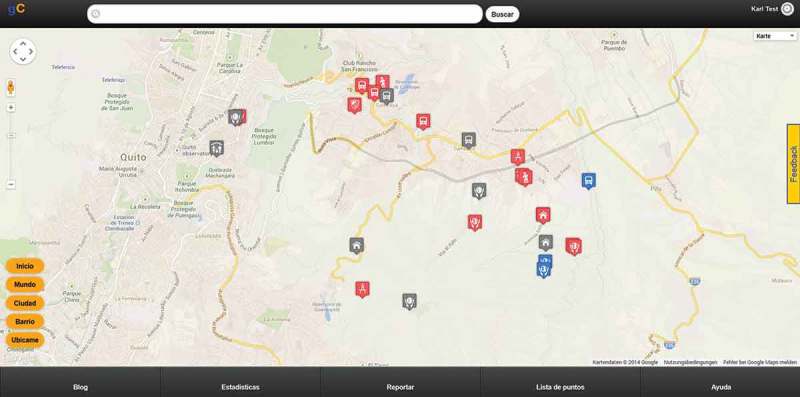
*GeoCitzen* platform prototype, snapshot.

The GeoCitizen-platform provides a framework to communities and their members for exchanging local knowledge, their everyday experiences, and observations about issues related to spatial (planning) processes, based on geographical features located on an online mapping platform (Figure 1). Observations that are reported by a *user* are structured into themes and categories. In these categories, the user provides detailed information about the observed subject for discussion in the neighborhood or community he/she is engaged with. The user can upload pictures, documents, and links to other sources on the web. After reporting, a problem-solving process can be started by specifying a process aim and the user can invite other *GeoCitizen* users or personal contacts to participate in the problem-solving process. If the user does not want to start the problem-solving process by him/herself, he/she can leave the reported problem as an observation, even if he/she wants to report as an anonymous user. A new observation can also be added to an already existing problem-solving process if the reporting user considers the observation fit for an existing process in the same thematic category or community.

Once one or more observations became object(s) of a problem-solving process, participating users can discuss the problem and possible solutions in a discussion forum. They can add more information and ask external experts for advice. The user who has initially started a problem-solving process assumes the role of a “*moderator*” of the discussion and formulates a proposal for the solution on which the participating users have to vote in order to approve the group’s decision. Users can join or leave the problem-solving process, according to their degree of concern about a topic, or invite other people to participate. In their solution finding process they are linked to user groups in other neighborhoods and municipalities that have the same necessities and that may already provide solutions for the same type of problem as best practice examples. The moderator is responsible to guide the group discussion and provide their members with additional information (expert knowledge about factors that drive spatial planning on larger scales) in order to keep up an active discussion and to support the finding of the best proposal for the problem-solving process.

**Figure 2.**
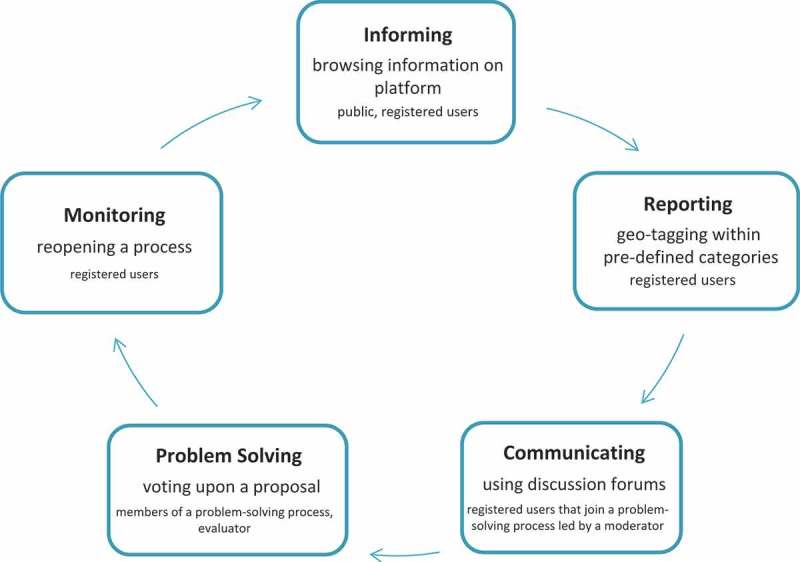
*GeoCitizen*-framework process flowchart.

Once the group confirms the proposed solution by voting, the result will be evaluated by an external expert in this topic (*evaluator*) who assesses whether or not the proposed solution is feasible. The *evaluator* supervises the ongoing solution finding and can support the group by giving advice or providing best practice solutions from similar processes in the same theme or category. The participants of the discussion process may or may not refer to the evaluator’s assessment when deciding upon a final solution for a problem. Finally, the identified solution can be presented to planning authorities, other community members and a broader public by traditional means of printed reports, a *GeoCitizen-blog-post*, by mail or through social networking platforms. In order to follow up on a finished process or a doubt whether or not appropriate measures for implementing a proposed solution have been taken, *GeoCitizen-users* can re-open an already finished process and can start a new discussion on the same topic by request to the evaluator (Figure 2).

If it is desired by the affected community, the proposed *GeoCitizen*-framework may include stakeholders from governments, municipalities, and other organizations which can amend the local knowledge stored in the system with information that simultaneously can be compared and analyzed by GIS-tools, and visualized as thematic layers on the platform. This should encourage ongoing discussions and the solution-finding process. Social cohesion and control in the affected communities should be boosted through active participation and the share of responsibility amongst their members in place of simply reporting problems to planning authorities. Hence, the *GeoCitizen*-approach is not a “pure” GIS, as it does not focus on a systemic approach to administrate spatial information as such. It is not a geo-locational service either, as it does not restrict itself to a mapping procedure of single events. It is not a “simple” social network, as it provides a well-organized framework for structured discussions and problem-solving processes instead of spontaneous and unstructured communication typical to social media. And it is not an online survey, as it does not focus on retrieving information in a unidirectional way.

**Figure 3.**
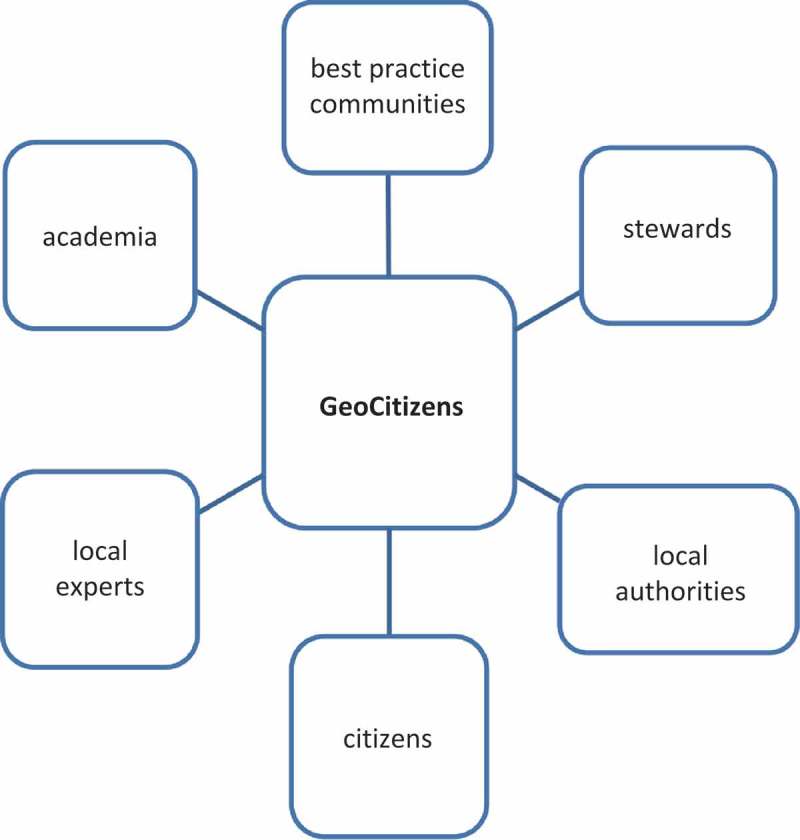
Potential stakeholders of the *GeoCitizen*-framework.

However, it combines elements of all these tools and approaches in one single and comprehensive framework, addressing stakeholders at different scales (Figure 3). At a local and neighborhood level, the *GeoCitizen*-framework addresses (1) local experts from community-based organizations (CBOs), NGOs, and citizen initiatives; (2) local authorities; and (3) ordinary citizens that are interested to make their voice heard in the process of improving their living environment. At a regional and global level, (1) experts from academia can contribute to a sound solution-finding process with expert knowledge (e.g. as evaluators), (2) other communities can provide best practice solutions for similar types of problems, and finally, (3) stewards can adopt a specific problem-solving process providing necessary funds or other incentives.

The *GeoCitizen*-platform is openly accessible for the public. Users, who not only want to browse published observations but also want to actively participate in a problem-solving process, must create a personal user profile and comply with specific terms of platform use. Users can report their observations within categories in which privacy issues are of specific interests (e.g. security) without revealing their user profile to the public. In order to track intended abusive use of the platform, the project team is monitoring the identity and behavior of participating users.

Following categories related to spatial planning are covered by the *GeoCitizen*-platform: land tenure and land use, public services and facilities, transport, environmental issues, public health, and security. Background data provide additional information about the status-quo and planned projects encompass cadaster and land value, zoning, topography and environment, natural and anthropogenic hazards, public infrastructure and facilities, census, and other socio-economic data.

## Pilot study

The proposed *GeoCitizen* approach is currently tested in a long-term study as part of a participatory land-zoning process that was initiated by the Municipal Government of the Metropolitan District of Quito, Ecuador (*Distrito Metropolitano de Quito – DMQ*) in spring 2013. This chapter focuses on an in-depth discussion of the land-zoning process, defining the participatory framework that provides the necessary foundation for applying the *GeoCitizen* platform. In addition, first insights into characteristics and patterns of platform use that are based on user experience of community members that tested the platform within in the pilot study are presented here.

This pilot study aims at providing replicable conclusions for making spatial planning more transparent and citizen orientated. It addresses deficits in spatial planning that are common in many developing and emerging countries of the Global South: for example, a lack of public services provided by governments, intransparent information policy about infrastructure projects, extraction and conservation of environmental resources, land tenure, and land use management. In these countries, traditional spatial planning has been discredited as it is often accounted for advocating inefficient, ineffective, and even illegal projects and inadequate service provision, resulting in a lack of legitimacy in the eyes of citizens (Rakodi 2001). Marginalized communities that have no or just little access to information and (political) decision networks are likely to be excluded from spatial-planning processes, as public expenditures are often not located to where they are needed most urgently or are not properly controlled by society (Resl 2006). As a consequence, conflicts between stakeholders in local and regional planning arise, mainly in following areas: (1) territorial management and public services, (2) public security, (3) public and individual transport, and (4) public (eco-) health management (Steinberg 2005; Fay and Morrison 2006; Freire 2006; Irazábal 2009; Rodgers, Beall, and Kanbur 2011). As a reaction to these deficiencies in traditional spatial planning, the collaborative management of conflicts constitutes a major focus of this pilot study by setting up a sound framework for participatory land-zoning processes and the application of the *GeoCitizen* platform.

## Pilot study area

The Capital District of Quito comprises an area of 4235 km^2^ and a population of 2.3 million people, which is 15.5% of the overall population of Ecuador (INEC 2011). It is divided in eight administrative zones that are organized in 32 urban, as well as 33 rural and suburban municipalities (*parroquias*). These municipalities are divided into neighborhoods (*barrios*) and communities (*comunas*). Whereas *barrios* are “ordinary” administrative subdivisions of municipalities, *comunas* resort to an ancestral organization and jurisdiction based on their indigenous heritage of communal land ownership and collective rights, ratified in the Constitution of the Republic of Ecuador of 2008 (Comisión Técnica de Aguas, Tierra, and Territorio y Comunas 2012). The history of these traditional communities goes back to the Colonial Era of Ecuador, when most of the fertile land in the Andean Highlands was settled by Spanish immigrants. A land reform in the 1970s passed some of these lands to the former indigenous servants (*huasipungueros*) and their communities (Costales-Samaniego 2006). Since 1993, *comunas* and *barrios* have been assigned new administrative competencies for managing areas of conservation in several administrative reforms by the national government.

The pilot study is carried out in communities and neighborhoods situated on or in vicinity of the Ilaló Mountain that is located between the inter-Andean Valleys of Tumbaco-Cumbayá and Los Chillos in the outskirts of Quito. The Ilaló is an extinct volcano ranging from altitudes between 2340 and 3180 m. Its heavily eroded surface is rugged by canyons (*quebradas*) that constitute the basins of the Chiche and San Pedro Rivers draining the central Andean Highlands of the Pichincha Province toward the Pacific Ocean (MECN 2010). These canyons are of major environmental importance, as they shelter remnants of the endemic Andean rainforest with a considerable number of endemic species that are extinct in wide parts of this highland ecosystem (FONAG 2008). Furthermore, the Ilaló provides important resources to the growing population that inhabits its slopes or the surrounding valleys, such as the provision of potable water and water for irrigation used in smallholder agriculture. In addition, the mountain is an important recreation area for the inhabitants of Quito and hence subject to increasing tourism activities (Bustamante 2012).

The Ilaló serves as a geographic barrier and island of biodiversity between the Valleys of Tumbaco-Cumbayá and Los Chillos. With a total population of approximately 400,000 people, both valleys have seen an explosion of housing and businesses activities over the last decade that dramatically increased the use of scarce land for housing, industry settlements, and the construction of transportation networks (World Bank 2008). In addition, agricultural activities (slash-and-burn) has severely degraded soils and interrupted ecological corridors on the mountain. In 2013, the new international airport of Quito was inaugurated in the Valley of Tumbaco-Cumbayá and is about to be connected to the City of Quito by an eight lane freeway (*Ruta de Integración de los Valles – Ruta* “*VIVA*”) touching the slopes of Ilaló, being heavily disputed in public (El Comercio 2013). During the last decade, especially commuters belonging to Quito’s growing middle class have settled down in gated communities accelerating urbanization of the former rural area. However, public authorities have not efficiently managed urban sprawl, land zoning, and the provision of public services in both valleys (Resl 2006). This increasingly courts the resentment of affected citizens and communities which feel to be excluded from decision-making processes by the established decision networks in politics and the private entrepreneurial (real estate) sector. Therefore, this area is considered as a representative case-study area for a first test-run of the *GeoCitizen* platform. Main partners in the case study are the University San Francisco de Quito (USFQ), CBOs, as well as representatives from administrative authorities (*barrios, comunas,* and *parroquias*) in the Valleys of Tumbaco-Cumbayá and Los Chillos acting as facilitators for coordinating participatory activities and promoting affiliated platform use.

## Participatory land-zoning process

In 1988, a total of 4813 ha of the Ilaló was declared as Protected Area (*Bosque y Vegetación Protectora*) by the Environmental Ministry of Ecuador. As a limit of the area of protection, the 2600 m contour line was determined (PSA-EMAAPQ 2006). Ever since, this area was subject of illegal settlements, waste disposal, and uncontrolled extraction of firewood and water used to irrigate land. In 2013, approximately 50% of the Ilaló was in (mainly agricultural) use. However, response of public authorities to these partly illegal activities was little, and isolated conservation initiatives of private and CBOs and individuals tended to be inefficient (Izurieta 2013). In 2006, the Municipal Enterprise of Potable Water and Waste Management (*Empresa Pública Metropolitana de Agua Potable y Saneamiento – EMAAPQ*) of Quito initiated the development of a land management plan for the Ilaló. Due to the opposition from lower administration levels and communities of the affected area, that were criticizing missing participation during the project, this plan never came into action (Penaherrera et al. 2010).

As a response to the increasing environmental problems related to growing population in the study area, the Municipal Government of Quito (DMQ) declared the Ilaló as one of the strategic areas of environmental conservation within its administrative boundaries in 2010. It commissioned the Council for Environmental Issues (*Secretaría del Ambiente*) and the Council of Territory, Habitat, and Housing (*Secretaría de Territorio, Habitat y Vivienda*) to initiate an evaluation of the state of conservation of the Ilaló. This evaluation aimed at identifying immediate actions for mitigating negative effects of urbanization and included the definition and delineation of so called Areas of Special Intervention and Recuperation (*Areas de Intervención Especial y Recuperación* – *AIER*) where spatial planning and conservation activities have to be carried out with a specific focus on specific environmental, socio-economic, and cultural conditions in the area (MECN 2010). The Ilaló was designated as such “Area of Special Intervention and Recuperation – AIER”, delineating its boundaries according to following parameters: (1) existing areas of endemic vegetation, (2) compatibility with the current land use plan of the Capital District of Quito (*Plan de Uso y Ocupación del Suelo* – PUOS), (3) vicinity to infrastructure projects (e.g. *Ruta* “*Viva*”), and (4) the connectivity of the drainage system with the main water basins of the Chiche and San Pedro Rivers (Izurieta 2013).

Intending to making spatial planning more transparent and citizen orientated,^1^ a participatory process was initiated by the Council of Territory, Habitat and Housing (*Secretaría de Territorio, Habitat y Vivienda*) of Quito in July 2013, in order to establish a new land use plan for the AIER Ilaló that should replace the land use plan in force for the Capital District of Quito (*Plan de Uso y Ocupación el Suelo* – PUOS). In a first step, a participatory land-zoning process was initiated. Based on its results, the *GeoCitizen* platform will be used to further socialize the proposed land use plan within the affected communities and to develop tangible actions and strategies for its implementation in a community-based land management plan. The first phase, the participatory land-zoning process for the AIER Ilaló, was concluded in September 2013. Representatives of all neighborhoods, communities, and suburban municipalities in the Valleys of Tumbaco-Cumbayá and Los Chillos, which territories share a part of the AIER Ilaló, were invited to participate in this process led by the community-based API. All participating representatives were previously appointed by their communities in town-hall meetings.

**Figure 4.**
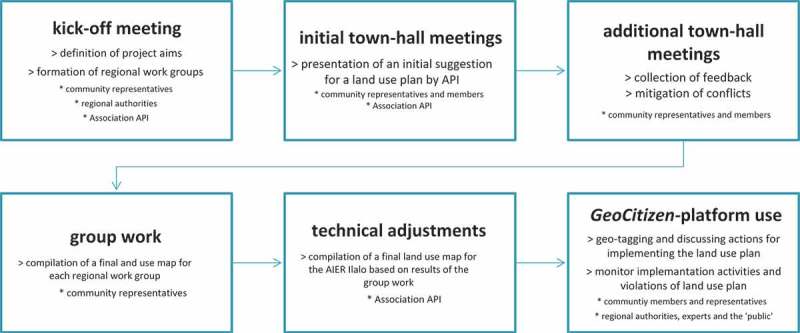
Participatory land-zoning process – *GeoCitizen*-Pilot Study Area Ilaló.

The participatory process was organized as following (Figure 4): In the first step, representatives of these communities; the Council for Environmental Issues (*Secretaría del Ambiente*); and the Council of Territory, Habitat, and Housing (*Secretaría de Territorio, Habitat y Vivienda*), as well as members of the *Association API* met in a kick-off meeting, where it was agreed upon objectives, procedures, and milestones of the process. Then, a total of four working teams (Workteam 1: Northern Slopes of Ilaló including the Barrio Pachosalas, Comuna Central, Comuna Leopoldo Chavez, Barrio Chiviqui, Comuna La Tola Chica; Workteam 2: Eastern Slopes of Ilaló: Barrios Olallaand Alcantarilla; Workteam 3: Southern Slopes of Ilaló: Comunas and Barrios of Parroquia La Merced and Parroquia Alangasi; Workteam 4: Southern Slopes of Ilaló: Comunas and Barrios of Parroquia Guangopolo) were formed, in which the representatives of communities geographically adjacent to each other joined together in order to work on a spatially coherent sub-zoning for their areas. For each territory assigned to the four working teams, members of the Association API presented a preliminary land use which was analyzed and discussed in town-hall meetings in each of the participating neighborhoods and communities. These preliminary land use plans are based on a technically feasible implementation of a total of six land use categories that have been predetermined by the Council of Territory, Habitat, and Housing (*Secretaría de Territorio, Habitat y Vivienda*) according to Ecuadorian law. These land use categories are: (1) Protection and Conservation (*Zona de Protección y Conservación*), (2) Recovery of Natural Vegetation (*Recuperación*), (3) Sustainable Residences (*Uso Sustentable de Vivienda*), (4) Sustainable Agroforestry (*Uso Sustentable de Agro-pecuario*), (5) Sustainable Tourism (*Uso Sustentable de Turismo)*, and (6) Service Areas (*Uso Especial*).

**Figure 5.**
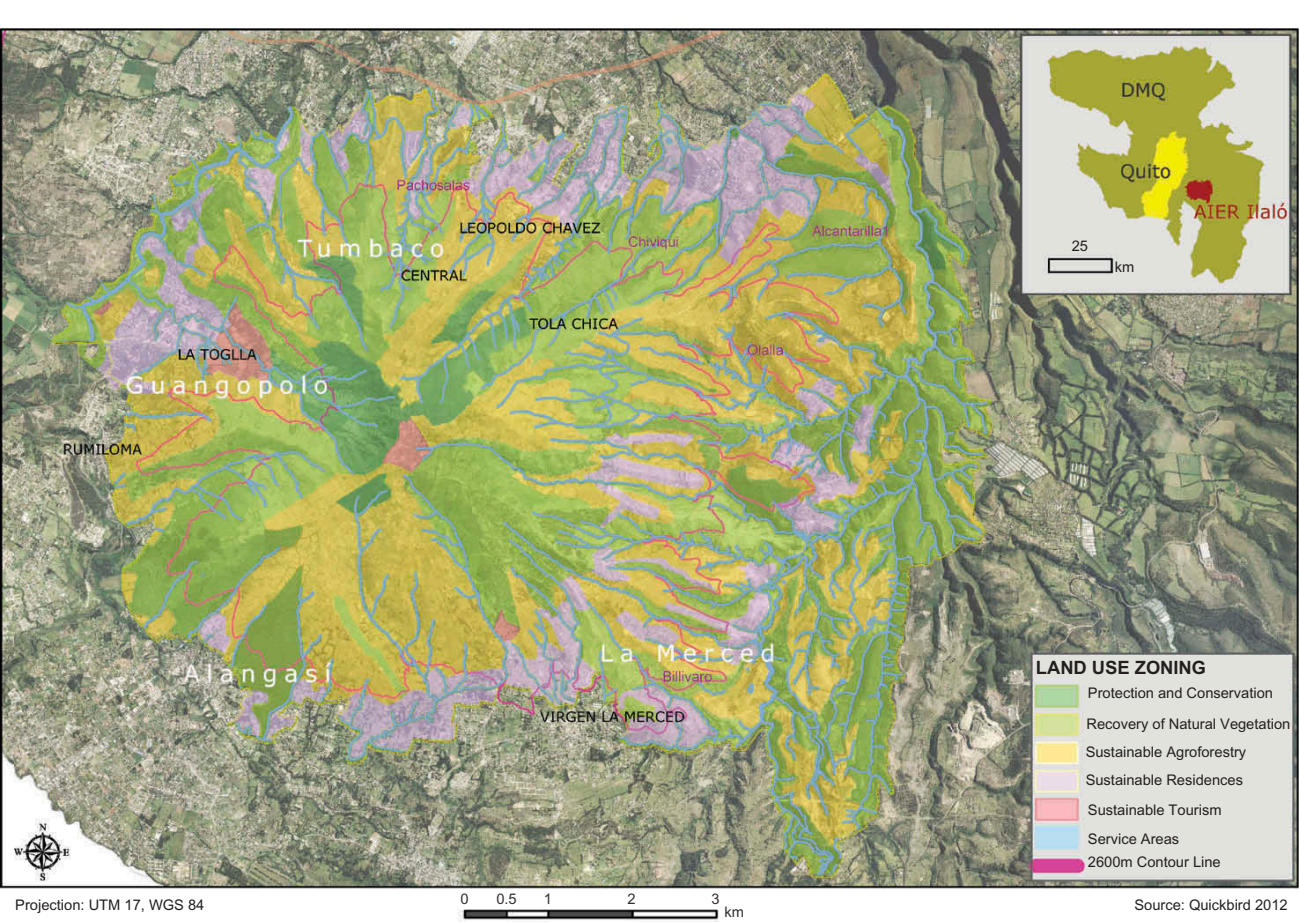
Preliminary results of the participatory-land zoning process, AIER Ilaló.

Feedback from participants of the town-hall meetings was immediately integrated to the plan, and diverging opinions were mitigated through in-depth discussions amongst the participants. In these meetings, community members were appointed for redesigning the preliminary land use plan according to the visions of their communities, collaborating with the other communities in their working team. During this phase, work was organized according to the local customs and legal frameworks of participation of each community, without any intervention from outside. By mid of September 2013, each working team handed in its revised land use plans as paper maps to the Association API, whose technicians digitized the paper maps and created a final version of the land use plan using ArcGIS-software. In areas where the maps proposed by the working teams showed spatially divergent allocation of land-use categories, they were matched according to technical criteria in order to provide one single land use plan for the whole AIER Ilaló (Figure 5).

Over all, representatives of five neighborhoods (the *barrios* Chiviqui, Olalla, Pachosalas, Alcantarilla, and La Merced), seven communities (the *comunas* Tola Chica, La Toglla, Central, Leopoldo Chávez, Virgen de la Merced, Alangasí, and Rumiloma) and three suburban municipalities (the *parroquias* La Merced, Alangasí, and Guangopolo) participated in the process. For those areas within the AIER Ilaló that did not participate, technicians of the Association API developed a preliminary zoning and integrated them into the overall land use plan. The participants attached specific importance to the fact that the suggested land use plan does not contradict the existing local land use/livelihood plans that have been previously elaborated by the participating neighborhoods or communities (*planes de manejo/planes de vida*). By the end of September 2013, representatives of all working teams handed over the final land use plan to the Council of Territory, Habitat, and Housing (*Secretaría de Territorio, Habitat y Vivienda*) of Quito. Upon a final proof by the council’s representatives, this plan should become legally binding for the AIER Ilaló in 2014.

After handing over the final land use plan to the authorities, community members within the study area will be invited to start using the *GeoCitizen* platform in order to discuss their ideas and define concrete actions how to implement the collaboratively developed land zoning. Users can access the platform in order to geo-reference what they think that should be done at a specific site for complying with the regulations of the plan. This can include for example initiating reforestation of degraded community land in areas that have been assigned to “Zone II – Recovery of Natural Vegetation” or suggesting and carrying out projects to promote local tourism in “Zone V – Sustainable Tourism”. Then, users can monitor the implementation of the collaboratively elaborated actions and projects, and can request experts, public authorities, or other stakeholders to provide and publish auxiliary data and information on the platform. In addition, conflicts regarding the implementation and monitoring of the land use plan (e.g. violation of the defined zoning through illegal housing or construction of infrastructure) can be reported online and shared with the community and the public in order to collaboratively elaborate transparent solutions including all affected stakeholders. As an overall objective, these reports and discussions should result in a participatory, transparent, and sustainable management plan for the study area.

## Preliminary insights

Simultaneously to carry out the participatory land-zoning process in the AIER Ilaló, the prototype of the *GeoCitizen*-platform (http://ilalo.geociudadano.org) was introduced to a set of potential platform users within the neighborhoods and communities in the pilot study area. These users addressed the research team searching for a tool that could help them to report, discuss, and monitor issues of interests in their communities taking advantage of the public and easy-to-access space of the internet. Workshops were organized in the communities (*comunas*) of Chiviqui, La Merced, and the neighborhood (*barrio*) Centro de Tumbaco, where the participants were instructed how to use basic platform functionalities in hands-on-sessions. Participants used the platform to report different issues: conflicts concerning land tenure and infrastructure planning (in specific regarding the construction of the “*Ruta VIVA*” freeway), the provision of public transport, and other types of public services, as well as environmental initiatives on the nearby Ilaló.

Using the ArcGIS for Server Javascript API, platform users could activate three different background layers in order to retrieve additional information for the pilot study area: the land use plan in force (PUOS), the proposed new participatory land use plan for the AIER Ilaló and a shapefile showing the proposed section of the *Ruta VIVA* freeway. This information was collected, prepared, and published by the research team in cooperation with the Association API.

Participants mainly were members of CBOs that have already been in contact with the research team during previous projects that were jointly carried out in their communities. However, also a (minor) number of citizens that were so far unknown to the research team participated in the workshops. Age, gender, ethnicity, and educational background varied. Furthermore, representatives of public authorities such as the suburban municipality (*parroquia*) Tumbaco-Cumbayá also participated in the workshops. Their attitude toward the *GeoCitizen* platform was ambiguous, ranging from consent and enthusiasm to mistrust. As a first step of concrete actions based on discussions on the *GeoCitizen* platform and in the accompanying workshops, community-based work (*mingas*) to identify wildfire risk spots, recuperate springs (*ojos de agua*), and to reforest a total of 15 ha of communitarian lands on Ilaló was initiated.

First insights of applying the *GeoCitizen* platform in the pilot study area suggest that this platform in specific and social-geoweb tools, in general, may be a key to initiate and structure discussion and planning processes, both on the community/citizens level and governmental level. Furthermore, feedback from platform users and participants in the pilot study support the assumption, that this approach may contribute to (1) mutual informing, (2) identifying and declaring local problem scopes and visions, (3) making local knowledge and initiatives available to a wider public, and (4) propose transparent decision making that suggests fair negotiations among stakeholders and leads to a monitoring process of conflicts and accomplishments toward the overall goals stated. However, experiences from the pilot study have shown that these tools should be integrated into existing decision-making structures in order to reach sustainable solutions. Simply publishing such tools on the internet for public use without embedding them in an accompanying participatory process, may neither encourage continuous and stable discussions between the stakeholders nor guarantee sustainable solutions for areas of action that have been identified by communities and their members.

Community work within the pilot study revealed that typical platform users are members of CBOs, who find great interest and incentives for their own causes adding “skills” and technical legitimacy to articulate their needs for penetrating traditional top-down planning processes of public authorities, and finding it convenient to publish their points of interest and concern on a web-based map. Following interest groups that announced a specific interest in applying the *GeoCitizen* platform within their communities addressed the research team during the pilot study: (1) the recently established *mancomunidad* La Merced/Alagansí/Guangopolo (an administrative union of the three mentioned *parroquias*), (2) the *Observatorio Ambiental de Quito* (a community-based initiative addressing environmental issues in Quito), (3) *Ciclopolis* (a local organization to promote urban cycling, sustainable transportation, and community building), as well as several communities and neighborhoods within an advanced process of social-media application for community-related communication.

However, still some stakeholders of major importance rejected the offer to participate in the participatory land-zoning process and the *GeoCitizen*-platform workshops. In particular, response from legal authorities of Tumbaco-Cumbayá, the suburban municipality with the highest population density in the pilot area, was negative – most probably due to interference of the real estate sector, who opposes the reorganization of the land-use plan (PUOS) in force. This attitude may resort to an existing mistrust of public authorities to present and transparently deal with sensitive information (e.g. a land use plan) in public. Missing participation by stakeholders from the public sector will result in a lack of information provided in a problem-solving process and may have negative implications for the quality of the identified solution. Hence, it is crucial to design the participation framework in a way that these actors realize the added-value of collaboration and the provision of information on the web, and giving them the opportunity to present their participation as part of a progressive and modern political agenda to a wider public. On a long run, best practice cases from other communities may help to convince reluctant stakeholders to participate. In addition, also public pressure may force those stakeholders to collaborate by dealing issues of common interest in the public space of the *GeoCitizen* platform. However, these preliminary observations give first indications of how geospatial web platforms (such as the *GeoCitizen* platform) might boost the collaboration and empowerment within spatial-planning processes. Still, this has to be further investigated in additional research in order to draw sound conclusions.

## Outlook

Usability issues have been identified as a major limitation for platform use amongst users with low ICT skills and little spatial literacy. Hence, the research team will focus on testing the *GeoCitizen* platform in an accompanying human computer interface-evaluation in order to gather user feedback that will be used for redesigning and recalibrating the platform. Experiences from these tests will be incorporated as guidelines in the development of the fully functional *GeoCitizen* platform.

In addition, the research team will aim at designing a specific framework for platform use, where facilitators who were previously appointed by the communities, support their members to access and use the platform to a full extent. Facilitators from the above-mentioned interest groups in the pilot study area will be trained in weekly seminars at the USFQ. They will promote platform use within their communities in order to attract a critical mass of users. Ideally, in each community, a permanent working station for using the *GeoCitizen* platform, that is accessible for community members that are not connected to the internet, is going to be installed. In addition, common social media such as Facebook, Twitter or Google+ will be integrated into the *GeoCitize*n platform, and user groups that already exist within the pilot-study area (e.g. the Facebook group *Ilaló Verde – Green Ilaló*) will be invited to use the platform.

In order to support decision-making processes with sound and unbiased information, background data about ongoing and planned (infrastructure) projects, topography and environment, as well as public infrastructure and facilities will be provided in the final version of the *GeoCitizen* platform by integrating spatial layers through the ArcGIS for Server Javascript API. Census data aggregated on a neighborhood level will be used to analyze typical user patterns in the pilot-study area. This systematic analysis will help to answer, whether or not the *GeoCitizen* approach and platform can help to make spatial-decision processes more transparent and democratically organized, and if this approach empowers citizens that so far have been excluded from spatial decision making, to make their voice heard. In particular, this research should encourage the development of a democratically legitimated and legally binding spatial management plan for the AIER Ilaló, and at best should be extended to other communities and municipalities in the area as best practice case. Final results of the long-term study are expected for autumn 2014.

## Note

1. The Ecuadorian Constitution of 2008 resorts to citizen participation (*participación ciudadana*) as an explicit constitutional power (*quinto poder*).
